# A 3D printing personalized percutaneous puncture guide access plate for percutaneous nephrolithotomy: a pilot study

**DOI:** 10.1186/s12894-021-00945-x

**Published:** 2021-12-24

**Authors:** Gao Keyu, Li Shuaishuai, Ashok Raj, Li Shuofeng, Liu Shuai, Zhang Yuan, Zhu Haitao, Wang Junqi

**Affiliations:** 1 Department of Urology, The Affiliated Hospital of Xuzhou Medical University, Xuzhou, 221000 China; 2 Xuzhou Medical University, Xuzhou, 221000 China; 3 Xuzhou Central Hospital, Xuzhou, 221000 China; 4 Xuzhou Children’s Hospital, Xuzhou, 221000 China

**Keywords:** Percutaneous nephrolithotomy (PCNL), 3D Printing technology, Puncture, Guide plate

## Abstract

**Objective:**

CT-Urography combined with 3D printing technology, digital design, construction of individualized PCNL puncture guides, and preliminary analyze their efficacy, safety puncture positioning for PCNL.

**Methods:**

Twenty-two patients with renal calculi were randomly selected at the affiliated Hospital of Xuzhou Medical University during 2017–2018. We randomly divided the patients into two groups: in 10 experimental groups, we used our 3D printing personalized percutaneous puncture guide access plate for PCNL, and in the control group, 12 patients with standard USG guide PCNL. The accuracy of puncture position, puncture time, and intraoperative blood loss was compared.

**Results:**

In the experimental group, 10 patients with 3D printing personalized percutaneous puncture guide access plate. The puncture needle was accessed through the guide plate and verified by the color Doppler. The single puncture, needle position, and depth success rate were 100.00% (10/10). The angles were consistent with the preoperative design. In the control group, 12 patients via USG guided PCNL success rate was 75.00% (9/12). The puncture time and amount of hemorrhage was (7.78 ± 0.94) min and (49.31 ± 6.43) mL, and (9.04 ± 1.09) min and (60.08 ± 12.18) mL, respectively. The above data of the two groups were statistically significant (*P* < 0.05).

**Conclusion:**

3D printing personalized percutaneous nephrolithotomy guide plate for PCNL can improve PCNL renal puncture channel positioning accuracy, shorten puncture time, reduce intraoperative blood loss, bleeding-related complications and provide a new method for PCNL renal puncture positioning, which is worthy of further clinical exploration.

## Introduction

Percutaneous nephrolithotomy (PCNL) is still the mainstream treatment for complex renal calculi. However, the complications such as bleeding, Infection, and residual stones during and after PCNL operation are the main burden needed more attention. Among them, bleeding is the most common complication, and the cause of bleeding is mainly the poor selection of renal puncture site and damage to renal vessels^.^ The establishment of the best skin-to-target renal puncture channel is a critical factor for successful operation. At present, the main preferable clinical methods of stone localization are X-ray (fluoroscopy) and Ultrasound [1, 2]. Fluoroscopy is a two-dimensional imagining system that lacks the stereoscopic sense of kidney tissue and stone location, with a low success rate of puncture and radiation damage [3]. Ultrasound can position calculi in multiple layers and effectively improve the accuracy of positioning. However, its low resolution and high requirements on ultrasonic technology of the surgeons limit its clinical application to a certain extent. 3D printing technology is a new technology developed in recent years. Using 3D printing technology to make personalized guide plates for different surgeries has been more and more applied in orthopedics and stomatology [4, 5]. However, this technology is still in the preliminary exploration stage in urology. The 3D printing plate can be implemented to avoid the damage of renal vascular puncture in the process of renal puncture localization and improve the accuracy of puncture localization. Through the 3D reconstruction model, doctors, patients, and their families can more clearly understand the stone localization, making the communication between doctors and patients more comfortable and has guiding significance for the planning of clinical surgery plans. In the experimental group, 10 patients who planned to undergo PCNL in the Affiliated Hospital of Xuzhou Medical University from 2017 to 2018 were selected. Using Computed Tomography Urography (CTU), combined with 3D printing technology and digital design, the individualized percutaneous nephrolithotomy guide plate was developed. The feasibility of its application in PCNL puncture localization was preliminarily discussed.

## Materials and methods

### General information

From 2017 to 2018, in the Affiliated Hospital of Xuzhou Medical University, a total of 22 patients with kidney stones were randomly selected for our study. All patients have signed informed consent forms. The experimental group (n = 10) had five males and five females. According to the stone side, there were 4 cases on the left side and 6 cases on the right side. According to the type of calculi, there were 5 cases of staghorn calculus, 2 cases of single calculi, and 3 cases of multiple calculi. Among them, lumbar pain and discomfort were found in 6 cases, an asymptomatic physical examination was found in 2 cases, gross hematuria in 2 cases. Two patients had a history of hypertension, and 4 patients had undergone extracorporeal shock wave lithotripsy (ESWL). The mean age was 48.60 ± 9.69 (28–62) years old. The control group (n = 12) had 7 males and 5 males. According to the stone side, there were 5 cases on the left side and 7 cases on the right side. According to the type of calculi, there were 4 staghorn calculi, 5 single calculi, and 3 multiple calculi. 7 cases complained of lumbar pain and discomfort, 3 were found asymptomatic on physical examination, and 2 were found gross hematuria. ESWL was performed in 5 patients. The mean age was 45.33 ± 8.52 (22–58) years old. There was no statistically significant difference in age, sex, stone side, stone type, and stone associated symptoms between the two groups (*P* > 0.05) (Table 1). Inclusion criteria: 1. All patients were diagnosed as renal calculi by CTU before surgery; 2. Surgical indications are consistent with PCNL [6]; 3. There was no significant disease of cardiopulmonary and other vital organ dysfunction; 4. Preoperative urine culture was negative, no urinary tract infection or Infection had been controlled; 5. No obvious bleeding tendency, normal liver and kidney function; 6. Invertebrate deformity and unable to prone patients; 7. communicate with patients and their families about the PCNL combined with 3D technology, digital design, and development of personalized percutaneous nephrolithotomy guide plate, obtain the consent of patients and their families, and sign the informed consent surgery.

**Table 1:** General information of two groups of patients

Patients profile	Experience group (n = 10)	Control group (n = 12)	*P*
Age: (years)	48.60 ± 9.69	45.33 ± 8.52	0.410
Stone side: (L/R)	4/6	5/7	0.937
Gender: (M/L)	5/5	7/5	0.696
Stone types: (staghorn/solitary/multiple occurrences (e.g.)	5/2/3	4/5/3	0.542
Complaint: (lumbar pain/discomfort/naked hematuria)	6/2/2	7/3/2	0.953

### A construction of 3D printing individualized percutaneous nephroscopy guides plate

**Fig. 1:**
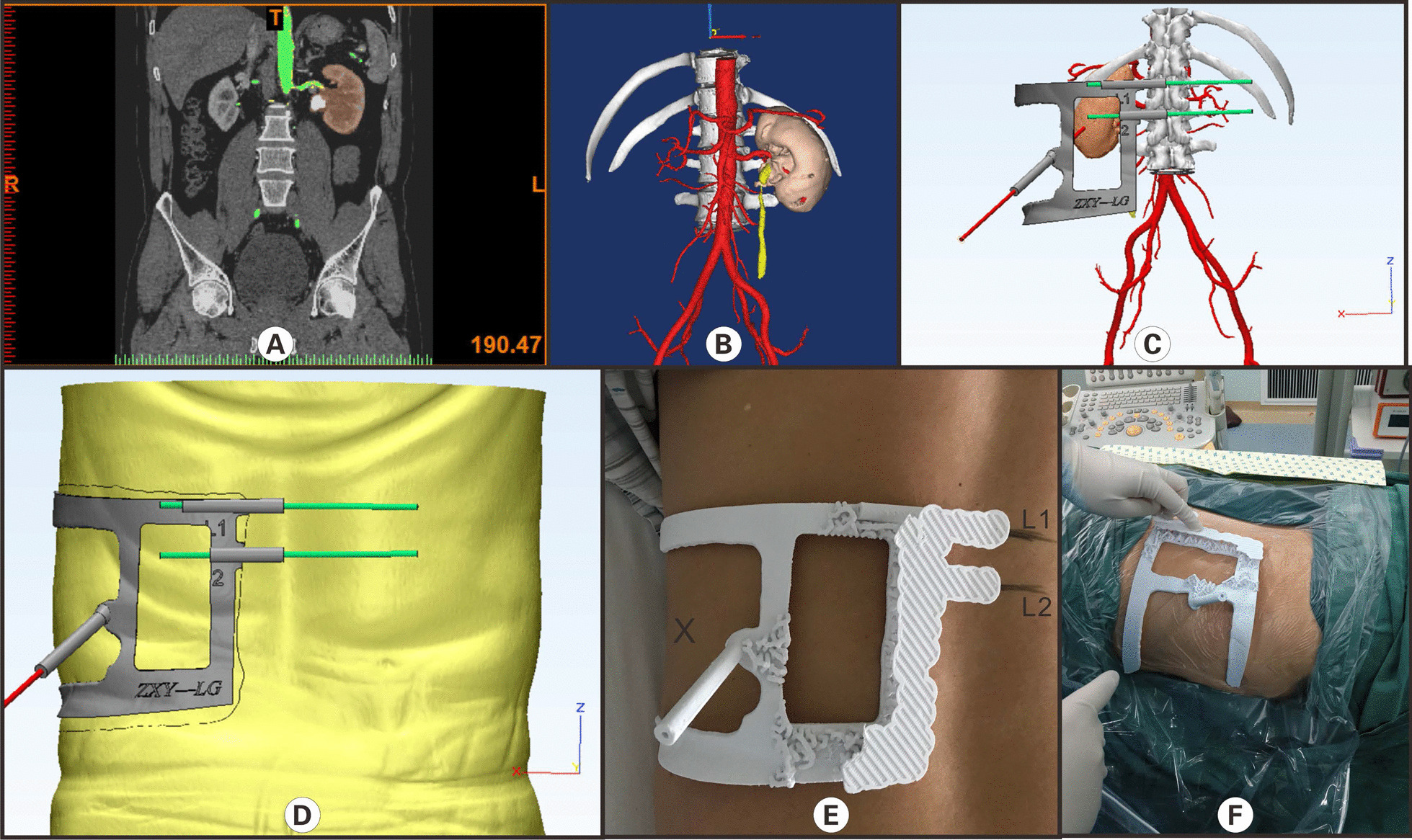
A Construction and application of a 3-D printing personalized percutaneous nephroscope puncture guide for a patient with left-sided kidney stones shown above. **A** CTU examination shows arterial phase and left kidney stone; **B** three-dimensional reconstruction of the kidney, renal vessels, and kidney stones was achieved by software through CTU images; **C** reconstructing the puncture location point according to the model, the angle, and depth of puncture, avoiding the main vessels; **D** designing the puncture guide plate that fits the skin; **E** 3D printed percutaneous nephroscope puncture guide plate was fixed on the patient skin. **F** The percutaneous puncture localized in patients with percutaneous nephroscope puncture guides by 3-D printing. (**Figure Legend** This figure is our own, drawn by Gao Keyu^1*^, Li Shuaishuai^2*^, and Ashok Raj^*^)

All 10 patients in the experimental group were placed in the supine position. CTU examination (256-slice spiral CT Philips) was performed with 5 mm thickness and 1.25 mm reconstruction. During a plain scan, the gray values of ribs and stones were higher than those of surrounding soft tissues, and the gray values of ureters, pelvis, and calyx were higher than those of renal artery, renal vein, aorta, and renal cortex; during arterial phase, the gray values of ureters, pelvis, and calyx were higher than those of renal artery, renal vein. During the excretion period, the gray value of the pelvis, calyx, and ureter can be increased because of the contrast agent to distinguish it from other soft tissues. The Dicom image file obtained by CT scan is used as initial data. Then, the Dicom file is imported into Mimics 17.0 software (Materialise Company of Belgium); according to the corresponding threshold segmentation and regional growth functions, kidney and pelvic calculi are established. 3-D reconstruction models of ribs and skin are saved in STL format. Then STL was imported into 3-Matic software, and the skin was smoothed with the guide plate. L1 and L2 were fixed points, and the lower edge of twelve or eleven ribs of the posterior axillary line were puncture points. Draw the surface surrounded by the positioning point, cut the surface appropriately, make the middle hollow, leave space for the color Doppler ultrasound probe, simulate the needle insertion cylinder to increase to 5 mm, use “Boolean subtraction” to determine the inner diameter of the needle insertion channel (1.5 mm), and generate the digital model of the guide. PLA material is used to print on the 3D printer (Raise 3D N2, Suzhou Wuchuang 3D Technology Co., Ltd.); after printing, the guiding device is used for reserve during operation after plasma sterilization (Fig. 1). Each guide plate costs about 500 USD. We also built the spinal model according to CTU, then designed the guide plate according to the position of the spinal model L1 and L2, and determined the point position of L1 and L2 on the guide plate. Before surgery, the patient was placed in a left decubitus position, and the patient grasped his hands over his knees to bend the spine. The surgeon found the bone marks on L1 and L2 body surfaces by hand touch and marked them on the body surface with a marker pen, respectively. During the operation, the position of the L1 and L2 guide plate was aligned with the marked on body surface to determine the position of the guide plate. During surgery, the guide plate is positioned with L1 and L2 markers aligned with L1 and L2 markers on the body surface.

### Position and surgical methods

Under general anesthesia, all 22 patients were placed in a prone position. Then, the surgical area was disinfected and draped. In the experimental group, we wear virtual reality glasses and can observe the 3D model established before the operation, better assisting the surgical puncture. During the operation, the patients' breathing is maintained by the ventilator. During the puncture, we will suspend the operation of the ventilator (within 1 min) or reduce the tidal volume of the ventilator to control the impact of breathing activities on the puncture. The X area in Figure E in Fig. 1 is reserved for the ultrasonic probe to monitor the depth and angle of the puncture needle in real-time. If any deviation, it can be adjusted in real-time After that, the sterilized puncture guide plate was fixed on the patient body. The assistant holds the puncture guide plate to prevent displacement. Then, the puncture needle was inserted through a puncture hole, and the angular position and accuracy of the puncture needle were assessed under USG. In the control group, a Puncture needle was inserted under the direct guidance of USG to locate a stone in the renal pelvis. Then, the PCNL procedure was performed in both groups.

### The accuracy of puncture (success rate of single needle puncture)

Intraoperative blood loss (intraoperative blood loss = Hb concentration in the sample of flushing fluid * volume of flushing fluid/preoperative Hb concentration [7]. Combined with intraoperative estimation), puncture time (puncture start to find the calyces where the stones are successfully located) were recorded.

### Statistical data analyzed by SPSS 20.0 software

The mean of measurement data (± standard deviation, *t* test, counting data (%), and Fisher exact test for statistical analysis. There was a significant difference in *P* < 0.05.

## Results

Before the operation, all ten patients were successfully designed and printed with a 3D percutaneous nephrolithotomy guide plate in the experimental group. The direction, depth, and angle of puncture were consistent with the design in the solid computer model. In all ten patients, the intraoperative puncture guide plate fitted to the patients' body skin, and the puncture needle under the guidance of the guide plate and verified by Ultrasound, the success of one puncture was 100.00% (10/10). In the control group, the success rate of one puncture was 75.00% (9/12), and there was no statistical significance between the two groups (*P* > 0.05). In both experimental and control groups, the puncture time and intraoperative blood losses were (7.78 ± 0.94/9.04 ± 1.09) min and (49.31 ± 6.43/60.08 ± 12.18) mL, respectively; there was a significant difference between the two groups (*P* < 0.05, Table 2).

**Table 2:** Comparison of puncture time, intraoperative bleeding, and puncture success of the first attempt between two groups

Parameter	Puncture time (min)	Blood loss (mL)	Successful puncture rate in the first attempt (%)
Experimental group	7.78 ± 0.94	49.31 ± 6.43	100.00 (10)
Control group	9.04 ± 1.09	60.08 ± 12.18	75.00 (9)
*T*	2.913	2.652	
*P*	0.010	0.017	0.221*

^*^Fisher Precise test

## Discussion

PCNL is an effective treatment for complicated kidney stones [8]. Previous studies have reported that PCNL stone clearance rate and surgical risk are closely related to the choice of the renal puncture site, and the establishment of the correct renal puncture channel is the premise to complete lithotripsy and reduce the incidence of complications such as intraoperative and postoperative massive hemorrhage, liver and pleural injury, and intestinal injury [9]. Therefore, the successful establishment of the renal puncture pathway is the main factor and difficulty of PCNL. We have researched and produced 3D printed guide plates to solve puncture positioning problems and increase puncture positioning accuracy and safety. The traditional methods of assisted renal puncture positioning mainly include X-ray or Ultrasound. Ultrasound and X-ray have their advantages and disadvantages. The X-ray can detect the whole process of a puncture in real-time and determine the residual stones after lithotripsy.

However, there is radiation damage to patients and surgeons, and the vertical depth of the stones is not feasible, and multiple puncture positioning may be required for patients with mild hydronephrosis [10, 11]. Ultrasound can distinguish the location of renal calculi and the thickness of surrounding renal parenchyma without radiation damage, thus improving the success rate of puncture and reducing the bleeding-related complications. However, Ultrasound requires the high ultrasonic skill of the surgeon and a long learning period, and it is not as straightforward as X-ray for stone imaging [12].

In this study, we found that the 3D percutaneous nephrolithotomy guide plate was made and applied to locate the kidney before puncture and guide the direction and angle of the puncture needle during puncture to reach the optimal renal calyx where the calculi were located, which could increase the accuracy of kidney puncture and simplify the puncture process and improve the puncture accuracy. Moreover, the 3D reconstruction model can enable patients and their families to a more precise understanding of the location of stones, specific surgical methods, and possible surgical risks compared with other imaging assistance techniques: which has a guiding significance for the planning of clinical, surgical plans, improve patients' trust in doctors, and makes the doctor-patient relationship more harmonious.

In this study, we designed an individualized percutaneous nephrolithotomy guide plate with 3D printing. L1 and L2 were selected as a bone landmark, and the puncture guide plate was reconstructed according to the scanning data of CTU in the plain scan period, arterial phase, and excretion period combined with PLA material. The thickness of the guide plate base is designed to be about 1 mm. The angle and depth of the puncture hole of the guide plate were selected according to the three-dimensional reconstructed renal vascular structure, the relatively large safety gap was selected, and the internal diameter of the puncture hole is designed to be 1.5 mm according to the type of the puncture needle. The excess internal diameter of the puncture hole would increase the puncture needle's instability and may cause the puncture angle deviation. The shape of the whole guide plate is designed as a long strip according to the bony marks and puncture points. The hollow design is adopted in part without support, which can save costs and leave space for the color ultrasound probe to monitor the puncture progress in real-time and assist positioning during puncture. We use PLA material because it has the characteristics of heat resistance, impact resistance, low cost, and low pollution, which can reduce the possibility of deformation of the guide plate in the progress of sterilization and disinfection. Under ultrasound-assisted verification during the operation, all the ten patients succeeded in one needle puncture (100.00%) under the guidance of a percutaneous nephroscope puncture guide plate. The positioning of the entry point, the depth, and the puncture angle were consistent with the preoperative design. Compared with the control group (75.00%), the accuracy of puncture location was significantly increased in the experimental group, and shortened the puncture time, and reduced the amount of intraoperative bleeding.

In the localization of renal puncture in PCNL surgery, we need to pay attention to uncontrollable factors such as (1) change in body position: the patients in the supine CT scan and prone position during surgery. (2) bony marker localization deviation. (3) respiratory activity. (4) intestinal or other intraperitoneal organ interference can reduce puncture accuracy and even lead to puncture failure. In order to minimize these uncontrollable factors, we will take the following measures in the future: (1) Preoperative CT scan position consistent with intraoperative position remain prone position and should be given abdominal augmentation according to PCNL position requirements, select the same hardness and flat operation beds because of the change of subtle body position will cause stones and surrounding tissue structure difference. (2) Before CT-urography, we can place two metal patches on the patients' body surface, design the puncture guide model according to the position of the two metal patches, and then keep the metal patches on the patients. During surgery, the marking points of the metal patch on the puncture guide plate are directly aligned with the metal patches on the patients' body surface, making the localization more accurate.

Although the above measures were taken to minimize the influence of uncontrollable factors, we believed that because preoperative intestinal preparation, intraoperative abdominal elevation, respiration, and other factors of the patient could not be consistent with preoperative conditions, there might be some deviation between the intraoperative renal position of the patient and the preoperative CTU, which could affect the puncture accuracy. In the past, 3D printing technology has been extensively used in orthopedics and maxillofacial surgery because of its bony structure, relative position is fixed, but percutaneous renal puncture for breathing is very demanding, mild kidney movement can affect puncture. Even if intraoperative breathing pause is used, the relative kidney position at this time cannot be guaranteed to be the same as that at preoperative CT examination. We preserve ample space for the ultrasonic probe under the puncture guide plate. During the puncture process, we can fine-tune the puncture angle in real-time and correct the puncture accuracy according to the images provided by the Ultrasound, which solves the above problems. However, the puncture template is already finalized and constructed; the puncture angle and position cannot be adjusted too much during the surgery. In the following improvement, we will connect the puncture channel and the base of the puncture template in the way of the ball joint, which is believed to solve this problem more perfectly. The error between clinical positioning of template and computer simulation can be reduced as much as possible through the above methods. In this paper, the 3D puncture guide plate relies on CTU, and CTU is related to patients' renal function. If patients' renal function is inferior, it may affect the CTU imaging, which is a limitation. We can consider combining various imaging techniques to establish a 3D model in context improvements, such as magnetic resonance urography. It should be emphasized that in the development of the model, we did not use ultrasound technology but only used Ultrasound to detect the position of the puncture needle for fine-tuning during the operation. The kidney is located behind the peritoneum. Considering the intestinal tract and other abdominal organs, we selected the puncture point below the 12th rib or around the 11th intercostal rib in the posterior axillary line. Then, the puncture access was determined in combination with the CTU scan results, and the position of the puncture needle was monitored in real-time by Ultrasound, which will minimize damage to the abdominal organs. At the same time, in the improved experiment, the patients' CTU examination position was taken as prone as possible to restore the operation. In addition, the puncture channel adopts a spherical joint, which can also avoid the injury of abdominal organs. As for the intraoperative placement of the guide plate, we had determined the bone marks on the patients' body surface before surgery and marked them with a marker pen. Therefore, we only needed to align the guide plate's L1 and L2 marker points with the body surface markers during the operation, and then the assistant helped to press the guide plate. The whole process does not take much time. With the development of 3D technology, we believe that these aspects will not impose restrictions on the clinical application of 3D printing technology in the urology department. In addition, another limitation of this paper is that the sample size is too small, which may affect our statistical result. Therefore, in the following work, we still need more samples to verify our conclusion.

## Conclusion

In summary, the 3D printing puncture guide provides a new method for PCNL puncture with high accuracy and safety compared with traditional color ultrasound puncture. This paper is a preliminary exploration of the application of 3D printing technology in a clinical puncture in urology. It is believed that with the maturation and development of 3D printing technology, it will be more prevalent in urology in the future.

## Data Availability

The datasets used during the current study are available on reasonable request from the corresponding author.
